# Clinical, functional, and quality-of-life characteristics of women with comorbid fibromyalgia and irritable bowel syndrome and their associations with FIQR scores

**DOI:** 10.3389/fphys.2026.1922718

**Published:** 2026-10-01

**Authors:** Antonella Bianco, Nicola Verrelli, Giuseppe Riezzo, Gaetana Laselva, Marco Grasso, Isabella Franco, Laura Prospero, Benedetta D’Attoma, Antonia Ignazzi, Maria Noemy Pastore, Elisa Grazioli, Attilio Parisi, Francesco Russo

**Affiliations:** 1 Laboratory of Movement and Wellness, National Institute of Gastroenterology IRCCS “Saverio de Bellis”, Castellana Grotte, BA, Italy; 2 Functional Gastrointestinal Disorders Research Group, National Institute of Gastroenterology IRCCS “Saverio de Bellis”, Castellana Grotte, BA, Italy; 3 Rheumatology Outpatient Clinic, National Institute of Gastroenterology IRCCS “Saverio de Bellis”, Castellana Grotte, BA, Italy; 4 Outpatient Pain Management Unit, National Institute of Gastroenterology IRCCS “Saverio de Bellis”, Castellana Grotte, BA, Italy; 5 Unit of Data Science, National Institute of Gastroenterology IRCCS “Saverio de Bellis”, Castellana Grotte, BA, Italy; 6 University of Rome “Foro Italico”, Rome, Italy

**Keywords:** comorbidity, exercise prescription, fibromyalgia, functional assessment, irritable bowel syndrome, quality of life

## Abstract

**Background:**

Fibromyalgia (FM) and irritable bowel syndrome (IBS) frequently co-occur, sharing pathophysiological mechanisms such as central sensitization and gut-brain axis dysregulation. Given that exercise is a first-line non-pharmacological intervention for FM, detailed baseline characterization may inform future exercise-based research. This study aimed to describe the physical, functional, symptomatic, and quality-of-life profile of women with FM+IBS comorbidity in relation to FM severity, assessed continuously and, secondarily, categorically using the Revised Fibromyalgia Impact Questionnaire (FIQR).

**Methods:**

This cross-sectional observational study, conducted at IRCCS “Saverio de Bellis” (Castellana Grotte, Bari, Italy), enrolled 33 women (mean age 47.7 ± 8.6 years) diagnosed with FM (2016 ACR criteria) and IBS (Rome IV criteria). Participants were stratified into three groups by FIQR score: Remission/Mild (n=7), Moderate (n=13), and High Severity (n=13). FIQR total score was analyzed as a continuous exposure using Spearman correlations with bootstrapped 95% CIs as the primary analysis; the three-group stratification was retained as a secondary, exploratory presentation. Assessments included pain (VAS), tender points, gastrointestinal symptoms (IBS-SSS), kinesiophobia (TSK), sleep quality (PSQI), physical activity (IPAQ), functional fitness tests, and quality of life (SF-36).

**Results:**

Perceived pain showed the strongest association with FIQR severity (Spearman’s rho=0.57, p<0.001), with values nearly doubling in the High Severity versus Remission/Mild stratum. Physical activity level (IPAQ; exact p=0.021) differed significantly across FIQR strata, with greater inactivity in the Moderate group. Lower-limb functional strength/endurance, handgrip strength, six-minute walk distance, kinesiophobia, and SF-36 Mental Component Summary were all significantly associated with continuous FIQR (all p<0.05); the Sit-to-Stand difference across categorical strata, significant under standard ANOVA (p=0.048), lost significance under Welch’s ANOVA (p=0.148) and Kruskal-Wallis testing (p=0.072). Gastrointestinal burden remained elevated across all groups, with no clear association with FIQR severity (rho=0.29, p=0.097). Sleep quality and SF-36 scores were markedly impaired regardless of FIQR class.

**Conclusions:**

In this exploratory sample, greater FM severity was primarily associated with increased pain and reduced lower-limb functional capacity, while gastrointestinal burden remained consistently elevated across the severity spectrum. These descriptive, hypothesis-generating findings may help guide the design of future, adequately powered studies addressing pain threshold, functional capacity, and gastrointestinal tolerance in exercise management of FM+IBS.

## 1 Introduction

Fibromyalgia (FM) is a chronic widespread pain syndrome that predominantly affects women, with a prevalence among adult women of between 3.6% and 5.6% (Marques et al., 2017; Kocyigit and Akyol, 2022). The clinical picture is underpinned by central sensitization mechanisms, which underlie the characteristic hyperalgesia and allodynia (Valera‐Calero et al., 2022). Pain, chronic fatigue, sleep disturbances, and physical deconditioning tend to reinforce each other in a vicious cycle that progressively reduces physical activity levels and participation in daily activities, thereby worsening overall disease severity over time (Lucini et al., 2024). This symptomatic burden is further exacerbated by comorbidity with irritable bowel syndrome (IBS), an association with FM that is now widely documented, statistically robust, and clinically relevant. Indeed, patients with IBS have an 80% increased risk of developing FM (Cole et al., 2006), in a bidirectional relationship that doubles the reciprocal probability of onset within five years (Erdrich and Harnett, 2025). Similarly, the incidence of IBS in patients with FM is 1.54 times higher than in the general population (7.47 vs. 4.42 cases per 1,000 person-years) (Yang et al., 2015).

This association is supported by shared pathophysiological mechanisms, including central sensitization, neuroinflammation, autonomic nervous system dysregulation, and gut-brain axis alterations (Elkalla et al., 2026), which have led some authors to propose that FM+IBS comorbidity may represent more than an additive overlap of the two conditions, potentially characterized by more frequent exacerbations, more severe gastrointestinal symptoms, and a more compromised quality of life compared to either condition in isolation (Tarar et al., 2023; Bheemaneni et al., 2025).

Exercise is widely recommended as the first-line non-pharmacological intervention for both conditions. Specifically, regular physical activity positively influences descending pain modulation, improves functional capacity, mitigates fatigue, and significantly enhances overall quality of life (Macfarlane et al., 2017; Johannesson, 2015). However, translating this recommendation into standardized clinical protocols remains complex, as the heterogeneity of clinical profiles makes it difficult to identify a universally valid, safe, and effective therapeutic approach. Despite clear clinical indications, individuals with FM and IBS notoriously exhibit low levels of physical activity.

In FM, kinesiophobia (the fear of movement) constitutes a primary barrier to initiating and maintaining structured exercise programs. Compared to healthy controls, patients with FM exhibit significantly higher levels of kinesiophobia, which translates into a marked reduction in physical activity: approximately 40% of these patients display systematic avoidance behaviors, with treatment adherence often compromised by pain intensity and its consequent impact on physical and social quality of life (Leon-Llamas et al., 2022; Karacay et al., 2023). Compounding this issue is the crucial role of sleep quality, which in FM represents not merely a consequence of the symptomatology, but an active pathogenic mechanism. Sleep deprivation, reported in up to 90% of patients (Hanuka et al., 2025), inhibits descending pain-modulatory pathways (Choy, 2015), triggering a synergistic effect that further lowers the exercise-tolerance threshold, even at equivalent levels of perceived pain (Bondesson et al., 2023).

A profile of reduced physical activity also characterizes patients with IBS, who often spontaneously restrict movement in an attempt to prevent or avoid the exacerbation of gastrointestinal symptoms (Costantino et al., 2022). In this context, proper exercise prescription is fundamental: inadequate training intensity or volume can worsen gastrointestinal symptoms, whereas moderate-intensity physical activity has been shown to reduce bloating and abdominal pain while improving psychological well-being (Johannesson et al., 2015; Machała-Ćwikła et al., 2024).

In individuals with functional disorders, and even more so when these present as comorbidities, an individualized approach to exercise, calibrated to the functional capacity and symptom burden of the individual patient, yields greater benefits compared to standardized protocols (Mazzorana et al., 2026). Recent reviews recommend precisely defining the type, intensity, frequency, duration, and progression of training according to individual needs (Han et al., 2024); however, such a strategy presupposes a detailed and multidimensional understanding of the baseline clinical profile. Based on these premises, the present cross-sectional observational study aims to describe the physical and functional characteristics, symptom burden, and quality of life in a sample of women with comorbid FM and IBS, in relation to disease severity assessed continuously and, secondarily, categorically using the Revised Fibromyalgia Impact Questionnaire (FIQR). We hypothesize that greater FM severity is associated with a more compromised multidomain clinical profile. As an exploratory, hypothesis-generating study, these findings are intended to inform the design of future, adequately powered research into individualized exercise prescription in this population.

## 2 Materials and methods

### 2.1 Study design

This cross-sectional observational study was conducted at the Movement and Wellbeing Laboratory of the IRCCS “Saverio de Bellis” in Castellana Grotte (Bari, Italy). Participants were recruited through collaboration among the “Functional Gastrointestinal Disorders” Outpatient Clinic, the “Rheumatology” Outpatient Clinic, and the “Outpatient Pain Management” Unit of the same institute, and by publishing an online questionnaire on the official website, inviting participation. The study was approved by the local Ethics Committee IRCCS Istituto Tumori “G.Paolo II” di Bari “Gabriella Serio” (protocol no.: 354/April 15, 2025) and was registered on ClinicalTrials.gov (NCT06923800). All participants signed a written informed consent form before being enrolled. The study was conducted in accordance with the Declaration of Helsinki and guidelines for good clinical practice.

The present analysis was not formally prespecified within the registered protocol of NCT06923800, which primarily concerns a subsequent exercise intervention. Recruitment took place between September 2025 and February 2026; baseline assessments were completed in March 2026.

### 2.2 Participants

Women with a concurrent diagnosis of FM and IBS were recruited. The inclusion criteria were: female gender; age between 30 and 65 years; a diagnosis of FM according to the 2016 American College of Rheumatology (ACR) diagnostic criteria (Wolfe et al., 2016), and a diagnosis of IBS according to the Rome IV criteria (Schmulson and Drossman, 2017). An additional criterion, willingness and motivation to undertake a structured physical activity program, was required for participation in the physical assessment battery and for eligibility for the subsequent registered exercise intervention (NCT06923800).

Exclusion criteria included: presence of other organic gastrointestinal conditions (inflammatory bowel disease, coeliac disease, intestinal neoplasms); clinically significant or uncontrolled heart conditions; active cancer or cancer in remission for less than five years; diagnosed major psychiatric disorders (psychosis, bipolar disorder, major depressive disorder in the acute phase); motor or orthopedic disabilities preventing the performance of field tests; and the use of analgesics, antidepressants/neuromodulators, hypnotics, or gastrointestinal medications that were initiated or modified within the four weeks before enrollment. Stable, long-term use of these classes of medications was permitted. The four weeks were chosen pragmatically to align with the recall interval used in previous studies, rather than on specific pharmacokinetic reasoning. Participants were asked to attend assessments while following their usual treatment regimen, without discontinuing or adjusting doses on the day of the assessment, and medication use was recorded via self-report at the initial visit.

### 2.3 Assessment

#### 2.3.1 Anthropometric characteristics

The study assessed various anthropometric parameters to investigate the subjects’ physical characteristics. The parameters included height, weight, and body mass index (BMI). Accurate measurements were obtained using a SECA 700 mechanical column scale and a SECA 220 altimeter (INTERMED S.r.l., Milan, Italy) to assess weight and height, facilitating subsequent calculation of BMI (kg/m^2^). Mid-upper arm, waist, and hip circumferences were not measured in the present study and are therefore not analyzed or reported here. Height and weight were measured by a single trained assessor following a standardized protocol (Esparza-Ros et al., 2019), in light clothing and without shoes, at a consistent time of day, with instruments periodically checked against a calibration reference.

#### 2.3.2 Fibromyalgia severity and pain intensity

The severity of FM was quantified using the FIQR, which assesses the condition’s impact on physical function, overall symptom intensity, and ability to perform daily activities (Bennett et al., 2009; Salaffi et al., 2013).

FIQR severity was stratified according to the thresholds established by (Salaffi et al., 2018): Remission ≤30, Mild >30 ≤45, Moderate>46 ≤65, High Severity >65, derived by the 75th–25th percentile reconciliation of adjacent ranks using the SF-36 as the external criterion. As the original thresholds leave a gap between 45 and 46, decimal FIQR scores falling within this interval were classified according to the following operational rule: scores from 45.0 to 45.5 were rounded down and classified as Mild, while scores from 45.6 to 46.0 were rounded up and classified as Moderate. This classification reproduces the observed FIQR range in each stratum exactly (Remission/Mild: 16.2–44.7; Moderate: 52.2–64.8; High Severity: 69.8–95.7). In the initial distribution, 1 participant fell into the Remission category (FIQR 16.2) and 6 into the Mild category (FIQR 39.3–44.7). Given the small size of the Remission group, the two categories were aggregated into the “Remission/Mild” stratum, which should not be interpreted as a validated FIQR severity category but rather as a methodological solution adopted for the analyses.

The number of tender points was assessed using standardized digital palpation at the 18 anatomical sites defined by the 1990 ACR criteria; the count of positive points (from 0 to 18) was used as a measure of the extent of widespread tenderness and symptom burden (Cott et al., 1992).

The subjective intensity of pain experienced in the past week was assessed using the Visual Analogue Scale (VAS), a continuous scale ranging from 0 (“no pain”) to 100 mm (“unbearable pain”), which has been extensively validated in populations with chronic pain (Bijur et al., 2001).

#### 2.3.3 Physical fitness

Physical fitness was assessed using a battery of five field tests, selected for their clinical applicability, low invasiveness, and documented validity in populations with chronic musculoskeletal conditions:

##### 2.3.3.1 Six-minute walk test

A validated clinical tool to assess submaximal exercise capacity and residual functional reserve, particularly suitable for sedentary or low-active patients. The protocol followed the ATS Statement: Guidelines for the Six-Minute Walk Test (2002), which requires participants to walk along a 30-meter indoor, straight, flat, and enclosed corridor at a brisk but sustainable pace, covering the maximum possible distance in 6 minutes, after which the distance walked is recorded (Enright, 2003). Participants were instructed to wear comfortable clothing and their usual athletic shoes. Heart rate was monitored before, during, and immediately after the test using a Polar OH1 heart rate monitor worn on a forearm strap and interfaced with an iPad operated by the technician, while perceived exertion was assessed using the Borg scale (Wilson and Jones, 1989). The operator informed participants of the elapsed time every minute and provided standardized verbal encouragement. The test could be temporarily paused at any time at the participant’s request: in such cases, timing was not suspended, but the duration and reason for the pause were recorded, in accordance with the ATS protocol (2002). The test was permanently terminated in the event of chest pain, severe dyspnea, dizziness, or at the participant’s request.

##### 2.3.3.2 Hand grip strength test

Maximal isometric grip strength was assessed using a digital dynamometer (CAMRY-Electronic Hand Dynamometer, model: EH101) (Pearn and Bullock, 1979; Huang et al., 2022). In a standing position, with the elbow flexed at 90° and the forearm in neutral position, each participant was instructed to perform a maximal voluntary contraction on the handle for approximately 3–5 seconds (Roberts et al., 2011). Standardized verbal encouragement was provided throughout the test. Self-reported hand dominance was recorded before testing, and the test was systematically initiated with the right hand. The protocol consisted of two trials per hand, with a 30–60-second rest interval between measurements. For analysis, the sum of the highest values recorded for each hand was used, as a study-specific decision, informed by the ACSM’s Guidelines for Exercise Testing and Prescription (Bayles, 2023), to provide a comprehensive measure of bilateral upper limb functional capacity.

##### 2.3.3.3 Sit-and-reach test

Lumbar and posterior chain flexibility was assessed using a standardized sit-and-reach box equipped with a centimeter-graded scale (Hoeger and Hopkins, 1992). Participants, without shoes, seated on the floor with legs fully extended and heels aligned with the edge of the box, were instructed to flex the trunk forward in a slow, controlled manner, keeping the knees fully extended and sliding both hands along the scale to the point of maximum tolerable excursion without bouncing. The zero point of the measurement scale was aligned with the vertical plane of the participant’s heels (foot-box contact line), in accordance with standard flexibility assessment guidelines (Bayles, 2023). The protocol consisted of two trials, with the best value (in cm) retained for analysis.

##### 2.3.3.4 30-second sit-to-stand test

Lower-limb muscle strength and endurance were assessed using the 30-second Sit-to-Stand test (Jones et al., 1999; Vaidya et al., 2017). Participants, seated in the center of a standardized chair (seat height: 43–45 cm, no armrests), with their back against the backrest and feet flat on the floor shoulder-width apart, were instructed to perform as many complete stand-sit cycles as possible within 30 seconds, keeping their arms crossed over the chest and refraining from using upper-limb support. A standardized demonstration and one familiarization trial were provided before testing. The total number of full stands (defined as achieving full hip and knee extension in the upright position) was recorded as the primary outcome.

##### 2.3.3.5 Single leg stance test

Static monopodal balance and postural stability were assessed using the Single Leg Stance Test under eyes-open conditions (Omaña et al., 2021). Participants, standing in their usual footwear on a rigid, flat surface, with their arms at their sides and the non-stance knee flexed at approximately 90° without contact with the supporting limb, were instructed to maintain the single-leg position for as long as possible, up to a predefined ceiling of 30 seconds. The protocol consisted of an initial trial for the dominant limb (self-reported by the participant): if the participant completed 30 seconds without error, the trial was considered valid and retained as the primary outcome; A single retest was permitted in the event of premature foot contact, loss of balance, or meeting termination criteria before 30 seconds; the best time achieved across both trials was retained for analysis (recorded to the nearest 0.1 second). A single retest, allowing for procedural or comprehension errors on the first attempt, is standard practice in balance-testing protocols. The test was terminated upon contact of the raised foot with the ground or supporting limb, displacement of the stance foot, uncrossing of the arms, or participant request. Testing in usual footwear and eyes-open conditions was selected to maximize ecological validity and minimize fall-related anxiety in a population with chronic pain and balance concerns.

All assessments were conducted in a controlled environment, at the same time of day, and by a single qualified operator (MSc in Preventive and Adapted Physical Activity Sciences). To minimize participant fatigue, the test battery was divided into two separate sessions. The 6MWT was administered on a dedicated day, given the substantial physical demand this test places on the study population. During the second session, tests were performed in a strictly standardized order: Single-Leg Stance Test, Handgrip Strength Test, 30-Second Sit-to-Stand Test, and Sit-and-Reach Test. A rest interval of 60–120 seconds was observed between tests to ensure adequate recovery, maximize performance, and prevent cumulative fatigue while minimizing assessment duration. Familiarization was limited to the single demonstration and practice trial previously described for the Sit-to-Stand Test; no separate familiarization trials were provided for the other four tests. No adverse events occurred during the assessments, and no participant was unable to complete the test battery. Finally, throughout the entire functional test battery, assessors were blinded to the participants’ FIQR severity classification at the time of testing, as FIQR scores were computed centrally after data collection.

#### 2.3.4 Physical activity levels

Physical activity levels were assessed using the Italian version of the International Physical Activity Questionnaire – Short Form (IPAQ-SF) (Mannocci et al., 2010). This self-report questionnaire consists of 7 items investigating the frequency (days/week) and duration (minutes/day) of vigorous-intensity activity, moderate-intensity activity, walking, and sedentary behavior over the past 7 days. Participants were categorized into three physical activity levels: low, moderate, or high, based on standardized cut-off criteria.

#### 2.3.5 Kinesiophobia

Kinesiophobia was assessed using the Italian version of the 13-item Tampa Scale of Kinesiophobia (TSK-13) (Monticone et al., 2010). This self-report questionnaire consists of 13 statements rated on a 4-point Likert scale ranging from 1 (*strongly disagree*) to 4 (*strongly agree*), with no reverse-scored items. Total scores range from 13 to 52, with higher values indicating greater fear of movement and (re)injury. The Italian adaptation demonstrates good psychometric properties, including adequate factorial structure and satisfactory internal consistency (Cronbach’s α > 0.70) in chronic pain populations.

#### 2.3.6 Health-related quality of life

Health-related quality of life (HRQoL) was assessed using the Medical Outcomes Study 36-Item Short-Form Health Survey (SF-36), a multidimensional questionnaire that measures eight domains: physical functioning, physical role limitations, bodily pain, general health, vitality, social functioning, emotional role limitations, and mental health. Scores for each domain range from 0 to 100, with higher values indicating a better quality of life. The SF-36 is one of the most widely used and validated tools for assessing HRQoL in FM, and is sensitive to differences in disease severity (Apolone and Mosconi, 1998). Physical Component Summary (PCS) and Mental Component Summary (MCS) scores were calculated using the norm-based scoring method of (Ware et al., 2001): each domain score was standardized as a z-score using reference population means and standard deviations (Physical Function 85.9 ± 21.8; Role-Physical 85.7 ± 23.2; Bodily Pain 75.3 ± 24.2; General Health 74.5 ± 19.7; Vitality 64.0 ± 18.4; Social Functioning 88.4 ± 20.5; Role-Emotional 93.7 ± 15.0; Mental Health 82.3 ± 13.2), and the eight domain z-scores were then combined using orthogonal factor score coefficients specific to the physical (PCS: 0.397, 0.367, 0.340, 0.150, 0.028, 0.050, -0.131, -0.225 for the domains in the order listed above) and mental (MCS: -0.160, -0.097, -0.123, -0.110, 0.257, 0.212, 0.390, 0.491) components, before transformation to a T-score metric (50 + 10 × aggregate z-score).

#### 2.3.7 Severity of gastrointestinal symptoms

The severity of IBS symptoms was quantified using the IBS Symptom Severity Score (IBS-SSS), a self-report instrument comprising five items referring to the past ten days: intensity of abdominal pain, severity of bloating, satisfaction with bowel movements, and degree of IBS interference with daily life, each rated on a 0–100 visual analog scale. The number of days with abdominal pain was instead recorded as the actual number of days (0–10) over the preceding 10 days, then multiplied by 10 to obtain the 0–100 component score. The total score (0–500) classifies patients as: mild (75–175), moderate (175–300), and severe (>300) (Francis et al., 1997).

#### 2.3.8 Sleep quality

Subjective sleep quality over the past month was measured using the Pittsburgh Sleep Quality Index (PSQI) (Buysse et al., 1989; Curcio et al., 2013), a 19-item self-report questionnaire that generates a total score (0–21) comprising seven components: subjective sleep quality, sleep latency, sleep duration, habitual sleep efficiency, sleep disturbances, use of hypnotic medication, and daytime dysfunction.

## 3 Statistical analysis

All statistical analyses were performed using Stata 18 (Stata Corp, College Station, TX, USA); continuous FIQR and bootstrap analyses were performed using Python 3 with SciPy (version 1.17.1) (Virtanen et al., 2020). Continuous variables are reported as mean ± standard deviation (SD); categorical variables are reported as frequencies and percentages.

The continuous FIQR total score was adopted as the principal exposure variable for all continuous outcomes. Associations between FIQR and each outcome were assessed using Spearman’s rank correlation coefficient, with 95% confidence intervals obtained by bootstrap resampling. Bootstrap resampling was performed on complete participant rows (list-wise), consistent with the absence of missing data in the analytic dataset (n=33 complete on all variables analyzed); a fixed random seed (42) was used for reproducibility. Reported correlation p-values are conventional (asymptotic) Spearman p-values; only the 95% confidence intervals, not the p-values themselves, were bootstrap-derived. Formal diagnostics for regression linearity and influential observations were not conducted for the reported slopes, which we note as a limitation.

As a secondary, exploratory analysis, participants were also stratified into the three FIQR-based severity categories described in section 2.3.2 (Remission/Mild, Moderate, High Severity) and compared using standard one-way ANOVA when the normality assumption (Shapiro-Wilk test) was met and variances were homogeneous (Levene’s test); Welch’s ANOVA when variances differed or group sizes were markedly unequal; and the Kruskal-Wallis test when normality was violated. For each outcome, a single omnibus test was selected in advance based on the distributional properties of that variable, and no pairwise *post-hoc* comparisons were performed or reported anywhere in this manuscript. Categorical variables were compared using the chi-squared test when expected cell counts were adequate, or a Monte Carlo exact test (200,000 permutations) when cell counts were sparse, as in the IPAQ comparison. A two-tailed p-value < 0.05 was considered statistically significant for all analyses. Given the exploratory nature of both analytical approaches and the conceptual overlap between FIQR-based grouping and several outcomes (section 2.3.2), all findings should be interpreted as hypothesis-generating rather than confirmatory.

For each categorical comparison, distributions were additionally inspected visually (dot plots by group) for skew and potentially influential values before selecting a summary statistic (mean ± SD versus median with interquartile range), given the limited diagnostic value of the Shapiro-Wilk test at n=7. A leave-one-participant-out sensitivity analysis was also performed on each categorical omnibus test, repeating the test with each participant removed in turn, to assess the stability of the result to individual observations; findings of this analysis are reported alongside the corresponding result in section 4.2.

### 3.1 Sample size and power considerations

No formal *a priori* sample size calculation was performed, as this study was designed as an exploratory, cross-sectional characterization of a clinically complex and poorly defined population. Rather than present a *post hoc* power calculation, which does not add information beyond the observed p-value and can be misleading (Hoenig and Heisey, 2001), we report bootstrapped 95% confidence intervals throughout (section 3) to convey the precision of our estimates directly. This limitation is consistent with the recognized recruitment challenges in populations with concurrent FM and IBS diagnoses, including clinical complexity, the requirement to satisfy multiple diagnostic criteria simultaneously, and documented reluctance to participate in research among patients with high symptom burden (Erdrich et al., 2020; Cardenas-Rojas et al., 2021). Accordingly, all findings should be interpreted as exploratory and hypothesis-generating rather than confirmatory, and replication in adequately powered cohorts is warranted before definitive clinical conclusions can be drawn.

## 4 Results

Of the 113 women initially identified through the recruitment network, 78 underwent eligibility screening; of these, 45 did not proceed to the baseline assessment (**Figure 1**). The most frequently reported reason was logistical or organizational constraints, such as transportation or scheduling difficulties (n = 12, 27%).

**Figure 1:**
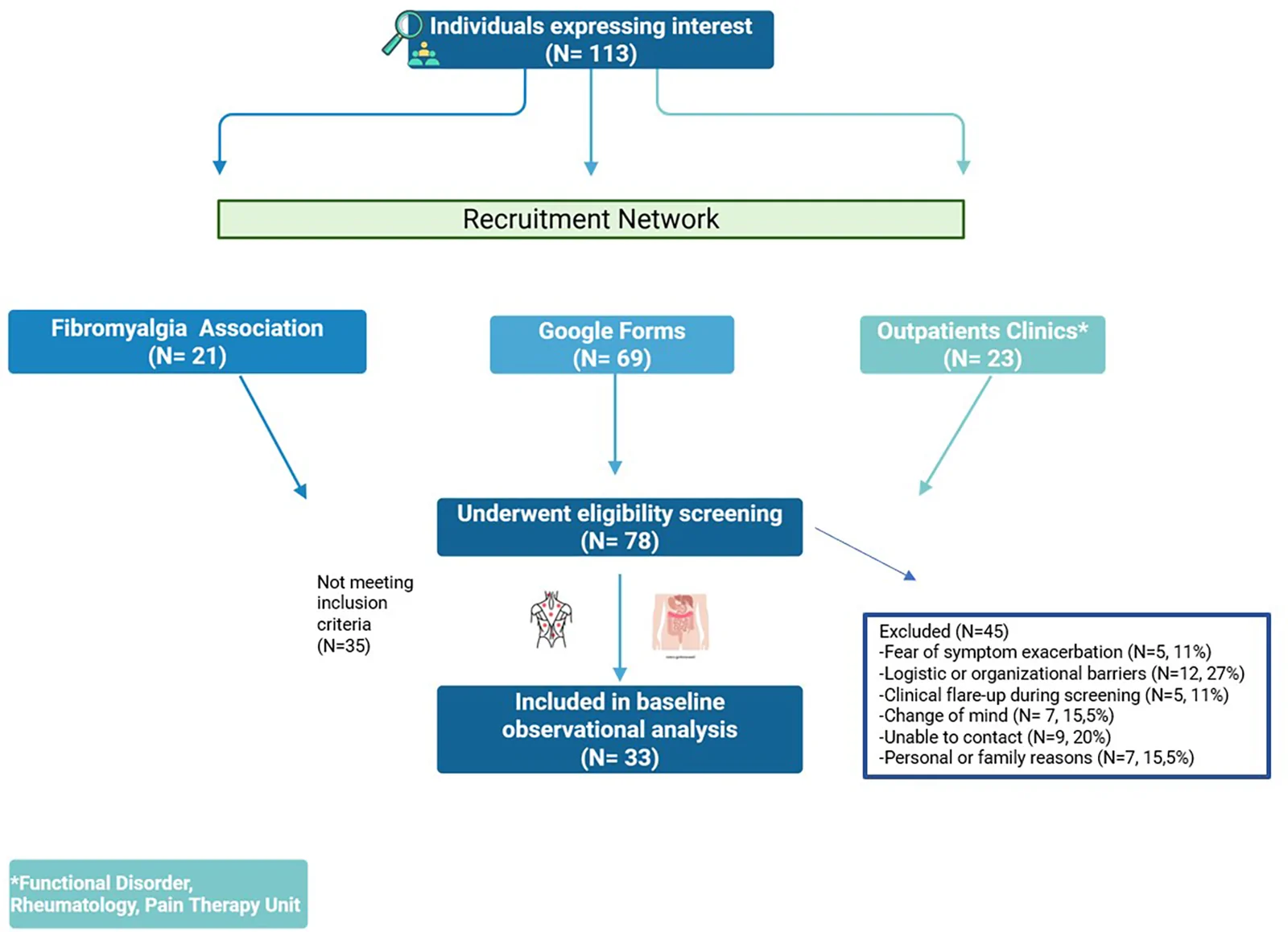
Recruitment flowchart. The diagram details the number of individuals identified, screened for eligibility, and included in the final analysis.

Thirty-three women with comorbid FM and IBS were included in the analysis. Based on FIQR scores, participants were divided into three severity groups: Remission/Mild, Moderate, and High Severity (**Table 1**). The three groups did not differ significantly in most sociodemographic characteristics, smoking status, alcohol consumption, or disease duration (**Table 1**); continuous BMI, however, showed a significant increasing trend across FIQR strata (ANOVA p=0.044; Kruskal-Wallis p=0.034) that was not apparent in the categorical obesity-prevalence comparison (p=0.179).

**Table 1:** Sociodemographic characteristics stratified by FM severity group (FIQR).

Characteristic	Remission/mild (n=7; 21.2%)	Moderate (n=13; 39.4%)	High severity (n=13; 39.4%)	P-value
Age (years)				
Age, years (continuous)	48.9 ± 6.7	49.3 ± 9.9	45.4 ± 8.4	0.49
< 40	1 (14.3%)	2 (15.4%)	3 (23.1%)	0.940
40–49	2 (28.6%)	5 (38.5%)	5 (38.5%)	
≥ 50	4 (57.1%)	6 (46.2%)	5 (38.5%)	
Years since FM diagnosis (mean ± SD)	6.9 ± 5.6	6.8 ± 5.0	7.2 ± 4.9	0.981
Education				
University degree	2 (28.6%)	2 (15.4%)	1 (7.7%)	0.814
Lower secondary	1 (14.3%)	2 (15.4%)	2 (15.4%)	
Upper secondary	4 (57.1%)	9 (69.2%)	10 (76.9%)	
Occupation				
Housewife	3 (42.9%)	3 (23.1%)	3 (23.1%)	0.644
Employee	3 (42.9%)	7 (53.8%)	4 (30.8%)	
Professional	1 (14.3%)	2 (15.4%)	3 (23.1%)	
Pensioner	0 (0.0%)	1 (7.7%)	3 (23.1%)	
Body Mass Index				
BMI, kg/m² (continuous)	23.8 ± 3.9	25.4 ± 3.8	28.3 ± 4.0	0.044
Normal weight	5 (71.4%)	8 (61.5%)	3 (23.1%)	0.179
Overweight	1 (14.3%)	3 (23.1%)	4 (30.8%)	
Obese	1 (14.3%)	2 (15.4%)	6 (46.2%)	
Smoking				
Never	5 (71.4%)	9 (69.2%)	12 (92.3%)	0.664
Occasionally	1 (14.3%)	1 (7.7%)	0 (0.0%)	
Current smoker	1 (14.3%)	3 (23.1%)	1 (7.7%)	
Alcohol intake				
Never	5 (71.4%)	9 (69.2%)	9 (69.2%)	0.969
Occasionally	2 (28.6%)	4 (30.8%)	4 (30.8%)	
Medication use				
Analgesics	1 (14.3%)	3 (23.1%)	5 (38.5%)	0.611
Antidepressants/neuromodulators	2 (28.6%)	3 (23.1%)	4 (30.8%)	1.000
Hypnotics/anxiolytics	0 (0.0%)	2 (15.4%)	0 (0.0%)	0.336
Gastrointestinal medications	1 (14.3%)	2 (15.4%)	4 (30.8%)	0.646

Continuous variables: mean ± SD; categorical variables: n (%). Medication classes were derived from self-reported current medication use recorded at study intake; percentages are not mutually exclusive, as participants could report medications from more than one class. Group comparisons used a Monte Carlo exact test (200,000 permutations) rather than the conventional χ² test, given sparse cell counts in some rows.

### 4.1 Association between continuous FIQR severity and clinical, functional, and quality-of-life outcomes (primary analysis)

As detailed in the Statistical Analysis, continuous FIQR total score was adopted as the principal exposure variable. Spearman correlations between FIQR and each outcome, with bootstrapped 95% confidence intervals, and the estimated difference in outcome per 10-point increase in FIQR, are reported in **Table 2a**. Perceived pain (VAS) showed the strongest association with FIQR (rho=0.57, 95% CI 0.23–0.78, p<0.001). Handgrip strength, six-minute walk distance, 30-second Sit-to-Stand performance, kinesiophobia (TSK), and the SF-36 Physical and Mental Component Summaries were all significantly associated with FIQR severity (all p<0.05). Tender point count, IBS-SSS, PSQI, sit-and-reach flexibility, and single-leg stance time showed no clear association with FIQR severity in this sample.

**Table 2a:** Association between continuous FIQR and clinical, functional, and quality of life outcomes.

Outcome	Spearman rho	p	95% CI (bootstrap)	Δ per 10-pt FIQR (95% CI)
VAS pain (mm)	0.566	**<0.001**	[0.23, 0.78]	7.46 [4.17, 10.49]
Tender points (n)	0.342	0.051	[-0.04, 0.66]	0.38 [-0.26, 0.92]
IBS-SSS total	0.294	0.097	[-0.09, 0.60]	12.63 [-2.27, 28.33]
TSK total	0.409	**0.018**	[0.04, 0.68]	2.12 [0.58, 3.06]
PSQI	0.261	0.142	[-0.11, 0.59]	1.17 [-0.09, 2.17]
Handgrip (kg)	-0.360	**0.040**	[-0.62, -0.01]	-2.23 [-3.58, -0.38]
Sit-and-Reach (cm)	-0.116	0.521	[-0.48, 0.27]	-0.12 [-2.15, 1.66]
Sit-to-Stand (rep/30s)	-0.413	**0.017**	[-0.68, -0.07]	-0.88 [-1.45, -0.32]
Six-Minute Walk (m)	-0.349	**0.047**	[-0.62, -0.03]	-11.62 [-24.59, -0.29]
Single Leg Stance (s)	-0.001	0.997	[-0.36, 0.34]	0.24 [-1.80, 1.90]
SF-36 PCS	-0.496	**0.003**	[-0.77, -0.14]	-2.31 [-3.60, -0.79]
SF-36 MCS	-0.413	**0.017**	[-0.67, -0.08]	-3.20 [-4.90, -0.96]

Spearman correlations between FIQR total score (continuous) and each outcome; 95% confidence intervals obtained by bootstrap resampling (5,000 resamples, percentile method); Δ per 10-pt FIQR = estimated difference in outcome per 10-point increase in FIQR (simple linear regression slope). n=33 for all rows. FIQR, Revised Fibromyalgia Impact Questionnaire; VAS, Visual Analogue Scale; IBS-SSS, Irritable Bowel Syndrome Symptom Severity Score; TSK, Tampa Scale of Kinesiophobia; PSQI, Pittsburgh Sleep Quality Index; IPAQ, International Physical Activity Questionnaire; PCS, Physical Component Summary; MCS, Mental Component Summary.

The values in bold indicate statistically significant results.

### 4.2 Symptom burden

Perceived pain intensity was the only outcome that differed significantly across the three categorical severity groups (p < 0.001), with a clinically substantial gradient between the Remission/Mild and more Moderate strata (**Table 2b**); as noted in section 4.1, this pain gradient is also reflected in the continuous FIQR association, and should not be read as independent confirmation of distinct severity profiles given the conceptual overlap between FIQR and pain (section 2.3.2). Tender point count and IBS symptom severity (IBS-SSS) did not differ significantly across the three categorical groups; kinesiophobia (TSK) likewise did not reach significance under the three-group comparison (p = 0.079), although it was significantly associated with FIQR when analyzed continuously (section 4.1).

**Table 2b:** Symptom burden and clinical assessments stratified by FM severity group (FIQR) — secondary, exploratory categorical presentation.

Outcome	Remission/mild (n=7)	Moderate (n=13)	High severity (n=13)	p (test)	Notes
VAS pain (mm)	37.1 ± 10.8	70.4 ± 15.1	74.6 ± 18.7	<0.001 (ANOVA)	ANOVA pre-selected (normal distribution, homogeneous variance)
Tender Points (n)	14.4 ± 3.5	16.0 ± 1.4	15.9 ± 3.5	0.486 (KW)	—
IBS-SSS total	286.4 ± 64.5	297.1 ± 65.8	320.0 ± 78.7	0.552 (ANOVA)	—
Total TSK	26.9 ± 8.8	29.0 ± 7.1	33.9 ± 5.9	0.079 (ANOVA)	—
PSQI	14.71 ± 6.26	12.62 ± 5.06	17.23 ± 5.31	0.112 (ANOVA)	—
Handgrip (kg)	49.2 ± 6.0	42.0 ± 13.2	40.8 ± 9.0	0.226 (ANOVA)	—
Sit-and-Reach (cm)	−1.7 ± 9.3	−2.2 ± 7.5	−2.7 ± 7.4	0.964 (ANOVA)	—
Sit-to-Stand (rep/30s)	13.1 ± 4.0	9.8 ± 2.9	9.6 ± 2.8	0.148 (Welch’s ANOVA, pre-selected)	Not significant under the pre-selected test; nominal significance under standard ANOVA (p=0.048) not stable to LOO removal (see Discussion).
Six-Minute Walk (m)	474.6 ± 57.7	435.9 ± 75.6	422.3 ± 57.7	0.245 (ANOVA)	—
Single Leg Stance (s)	24.67 ± 6.70	18.54 ± 10.67	20.15 ± 9.45	0.394 (ANOVA)	—
IPAQ (Low/Mod/High %)	14/29/57	69/31/0	54/8/38	0.021 (Monte Carlo exact, sole reported test — conventional χ² inappropriate, cells <1)	
SF-36 PCS	39.6 ± 10.8	33.7 ± 5.2	31.9 ± 6.9	0.123 (KW, pre-selected)	Non-normal distribution in one stratum; KW selected in advance.
SF-36 MCS	26.2 ± 13.5	17.1 ± 13.1	15.0 ± 13.4	0.086 (KW, pre-selected)	Non-normal distribution in two strata; KW selected in advance.

Continuous variables: mean ± SD; categorical variables: n (%). The single omnibus test used for each row is indicated in the table (selected in advance based on the distributional properties of that outcome); no pairwise *post-hoc* comparisons are reported in this manuscript. FIQR, Revised Fibromyalgia Impact Questionnaire; VAS, Visual Analogue Scale; IBS-SSS, Irritable Bowel Syndrome Symptom Severity Score; TSK, Tampa Scale of Kinesiophobia; PSQI, Pittsburgh Sleep Quality Index; IPAQ, International Physical Activity Questionnaire; PCS, Physical Component Summary; MCS, Mental Component Summary. Scale ranges, units, and score direction for each row: VAS pain, mm, 0–100, higher = worse; Tender Points, count, 0–18, higher = worse; IBS-SSS, points, 0–500, higher = worse; TSK, points, 13–52, higher = worse; PSQI, points, 0–21, higher = worse; Handgrip, kg, higher = better; Sit-and-Reach, cm, higher = better; Sit-to-Stand, repetitions/30s, higher = better; Six-Minute Walk, meters, higher = better; Single Leg Stance, seconds (ceiling 30s), higher = better; SF-36 PCS/MCS, T-scores (mean = 50, SD = 10, referenced to the general population), higher = better.

The IBS-SSS remained elevated across all three groups; no statistically significant difference was detected across the categorical strata (p = 0.552), and no clear association was found with continuous FIQR (rho=0.29, p=0.097, section 4.1). We interpret this as an absence of clear evidence for an association in this sample, not as evidence that gastrointestinal burden and FM severity are independent.

Sleep quality, assessed using the PSQI, was severely impaired across all severity groups (all mean scores > 10, well above the clinical cut-off of 5), with the highest values recorded in the High Severity group (17.23 ± 5.31), though inter-group differences did not reach statistical significance (p = 0.112).

Physical activity levels, measured by the IPAQ-SF, differed significantly across groups (Monte Carlo exact p = 0.021, in place of the conventional chi-square test given sparse cell counts): the Remission/Mild group showed the highest proportion of highly active participants (57.1%), while the Moderate group was dominated by low-activity levels (69.2%); notably, the High Severity group displayed a bimodal distribution between low (53.8%) and high (38.5%) activity categories, suggesting a heterogeneous behavioral response to symptom burden in the most severely affected patients.

### 4.3 Physical fitness

Among the fitness measures compared categorically, the Sit-to-Stand test reached statistical significance under standard one-way ANOVA (p = 0.048); this result did not, however, hold under the more conservative Welch’s ANOVA (p = 0.148) or Kruskal-Wallis test (p = 0.072), despite homogeneous variances (Levene’s p = 0.78) and normal distributions in all three groups. This is a fragile result: the categorical Sit-to-Stand comparison does not survive Welch’s ANOVA or the Kruskal-Wallis test, whereas the continuous-FIQR association for the same test held (rho = -0.41, p = 0.017; section 4.1) and better reflects a genuine reduction in lower-limb functional strength/endurance with increasing FM severity. A leave-one-participant-out sensitivity check reinforces this: 23 of the 33 possible single-participant removals flip the categorical result across the p=0.05 threshold, indicating that the standard ANOVA finding (p=0.048) is not stable to the exclusion of individual participants.

The Single Leg Stance Test, a measure of static postural balance, showed the best performance in the Remission/Mild group (24.67 ± 6.70 s) compared to the Moderate (18.54 ± 10.67 s) and High Severity (20.15 ± 9.45 s) groups, though this difference did not reach statistical significance (p = 0.394). Ceiling performance (30 s) on this test was reached by 3/7 (42.9%) participants in the Remission/Mild group, 5/13 (38.5%) in the Moderate group, and 5/13 (38.5%) in the High Severity group.

The remaining categorical comparisons for handgrip strength and Six-Minute Walk distance did not reach statistical significance across the three groups, although both were significantly associated with FIQR when analyzed continuously (section 4.1); Sit-and-Reach showed neither a significant categorical difference nor a continuous association with FIQR.

### 4.4 Health-related quality of life

Both SF-36 component summaries showed a descending gradient with increasing FM severity; the categorical difference in the Physical Component Summary did not reach statistical significance (p = 0.088), though both PCS and MCS were significantly associated with continuous FIQR (section 4.1). Regardless of group, all mean scores were markedly below established population norms, reflecting a pervasive impairment in health-related quality of life across the entire sample. The SF-36 eight-domain scores were analyzed in relation to FM severity and are reported in Supplementary **Supplementary Table S1**. Six of the eight domains, Physical Function, Bodily Pain, General Health, Vitality, Social Functioning, and Mental Health, differed significantly across the three FIQR-based groups (all p<0.05 under standard ANOVA, Welch’s ANOVA, and Kruskal-Wallis) and showed significant Spearman correlations with continuous FIQR (rho range -0.40 to -0.66, all p<0.05). Role-Physical and Role-Emotional showed neither a significant categorical difference nor a significant continuous association.

## 5 Discussion

This cross-sectional study characterized the baseline multidimensional profile of women with the FM+IBS comorbidity in relation to disease severity, as measured by the FIQR, using continuous FIQR as the principal exposure and three-group categorical stratification as a secondary presentation. The results only partially support our initial hypothesis, outlining a complex clinical picture. Specifically, increasing FIQR severity was associated with greater perceived pain intensity, reduced lower-limb functional capacity, reduced handgrip strength and walking distance, greater kinesiophobia, and poorer mental health-related quality of life. Conversely, the burden of gastrointestinal symptoms, sit-and-reach flexibility, and postural balance showed no clear association with FM severity in this sample.

The strongest association observed in our data was between continuous FIQR and perceived pain scores, also reflected in the categorical comparison (Remission/Mild scores just above the clinical threshold for moderate pain, versus values indicative of intense and functionally limiting pain in the Moderate and High Severity strata). Because FIQR itself incorporates pain-related items, this association is expected to some degree and should not be over-interpreted as an independent finding; nonetheless, it is consistent with the established role of baseline pain intensity as a determinant of exercise tolerance and adherence in FM more broadly (Nunan et al., 2022; Adams et al., 2023). We therefore consider it plausible, though not directly demonstrated in this cross-sectional dataset, that more severely affected patients may benefit from a more gradual exercise approach emphasizing low-impact activities. This hypothesis would need to be tested in an interventional design.

No significant differences in tender point counts were observed among the groups, nor was there a clear association with continuous FIQR scores. Tender point responses to pressure are influenced by multiple factors beyond peripheral nociceptor sensitization. Because neither tender point counts nor VAS pain scores directly assess central sensitization, the discrepancy between elevated pain intensity and stable tender point counts is consistent with, though not definitively confirmatory of, the central sensitization model of FM (Valera‐Calero et al., 2022). From a mechanistic standpoint, structured exercise remains a plausible intervention across all severity levels, as it promotes the activation of descending inhibitory pain pathways. It should be noted, however, that this specific mechanism was not directly measured in the present study (Quodling et al., 2025).

One notable finding is the absence of a clear categorical or continuous association between gastrointestinal symptom severity and FM severity. IBS-SSS scores were consistently elevated, falling within the moderate-to-severe range across all groups. We interpret this as an absence of clear evidence for an association between gastrointestinal burden and musculoskeletal FM severity in this sample, rather than as proof that the two are unrelated, a distinction that matters given our limited sample size and the width of the corresponding confidence interval.

The observed pattern suggests that gastrointestinal symptom burden and fibromyalgia impact may not change in parallel, although the estimate was imprecise and requires confirmation in larger samples. This aspect may warrant consideration in the design of future exercise intervention studies, which should evaluate whether a personalized approach integrating gastrointestinal symptom assessment could optimize the intensity, mode, and progression of the intervention (Costantino et al., 2022). Relying solely on the FIQR score risks underestimating the gastrointestinal component, even in patients with mild-to-moderate FM.

Among the five functional assessments, the 30-second Sit-to-Stand test showed a statistically significant categorical difference under standard ANOVA, though not under Welch’s ANOVA or the Kruskal-Wallis test; it did, however, show a significant association with continuous FIQR, which we consider evidence of an inverse cross-sectional association between continuous FIQR and lower-limb functional strength and endurance. A leave-one-participant-out check further supports this reading: the categorical result is not stable to the removal of individual participants, whereas the continuous association remained statistically significant (p<0.05) after removal of any single participant (33 of 33 leave-one-out removals), supporting its relative stability compared with the categorical result.

This result is consistent with the pathophysiology of FM, in which chronic pain, physical inactivity, and muscular deconditioning disproportionately affect the antigravity muscles (Quodling et al., 2025). The performance recorded in the Moderate and High Severity groups (approximately 9–10 repetitions/30 s) is lower than normative values reported for the general adult population, indicating a functionally significant deficit (Panton et al., 2006; Álvarez-Gallardo et al., 2019).

The lack of categorical statistical significance for the remaining functional assessments does not rule out the presence of clinically relevant differences and, for handgrip strength and 6MWT distance, is resolved when FIQR is analyzed continuously, where both show significant associations; single-leg stance and sit-and-reach showed no clear association with FIQR under either approach.

Our results are consistent with prior literature showing that lower physical fitness levels correlate with greater FM severity (Aparicio et al., 2013; Soriano-Maldonado et al., 2015). These exploratory findings may inform future studies evaluating multicomponent exercise programs incorporating aerobic, strength, and balance training.

Kinesiophobia (TSK) was significantly associated with continuous FIQR severity, although the categorical three-group comparison did not reach significance. Scores in the High Severity group approached the threshold of 37, which is commonly used as a cutoff for clinically significant Kinesiophobia (Neblett et al., 2016).

This finding suggests that in the most severe presentations of FM, the psychological component of fear of movement may serve as an additional barrier to exercise participation, supporting the integration of gradual movement-exposure strategies into personalized protocols for this patient subgroup.

In accordance with established literature, health-related quality of life showed a decreasing trend in line with FM severity, yet confirmed a well-known pattern: absolute scores consistently fall below normative thresholds across all severity levels (Salaffi et al., 2009). Both SF-36 component summaries were significantly associated with continuous FIQR, reinforcing this pattern beyond the categorical trend originally reported. At the domain level, six of the eight SF-36 domains followed the same pattern as the PCS/MCS summary scores discussed above, while Role-Physical and Role-Emotional showed no clear association with FM-related impact, likely reflecting the well-documented floor/ceiling effects of these two dichotomous role-limitation items in SF-36 scoring.

Sleep quality was severely compromised across the entire cohort, with PSQI scores in all groups far exceeding the clinical threshold of 5. This aligns with the well-established bidirectional relationship between pain and sleep in FM (Bigatti et al., 2008; Moldofsky, 2008). We found no statistically significant difference in PSQI across FIQR strata, which is consistent with, though does not by itself establish, sleep quality as a factor that is uniformly impaired across the FM+IBS severity spectrum rather than one that tracks FM severity specifically.

An unexpected but statistically significant finding emerged from the assessment of physical activity levels. The Remission/Mild group showed a surprisingly high percentage of participants classified as highly active, while the Moderate group was predominantly characterized by low activity levels. In subjects with fibromyalgia, the subjective perception of exertion and the ability to distinguish between moderate and vigorous activity intensities may be compromised by altered interoceptive processing and central sensitization (Borg et al., 2018). Consequently, self-reported physical activity data (e.g., via the IPAQ) are susceptible to systematic bias in this clinical population. This interoceptive/central-sensitization explanation remains speculative; IPAQ measurement error, the small and unequal sizes of the severity strata, and sampling variability represent equally plausible, non-mutually exclusive alternative explanations for this pattern. Future studies should ideally supplement self-reported physical activity data with objective monitoring, such as accelerometry, to strengthen the assessment of activity levels in this population (McLoughlin et al., 2011).

The data from this study are part of a broader research program aimed at describing the comorbidity between FM and IBS from various clinical perspectives. Previous studies by our group have documented, in this population, a psychological profile characterized by high levels of somatization and distress, reduced quality of life, and greater impairment of the intestinal barrier compared with patients with isolated FM or IBS (Prospero et al., 2025; Russo et al., 2025). These studies, like the present one, are cross-sectional in nature and do not aim to define FM+IBS as a clinically distinct phenotype. Rather, this study serves as a further descriptive characterization within the same line of research, extending the analysis to the functional and physical domains to provide an empirical basis for future studies on personalized exercise prescriptions in this population.

## 6 Limitations and future prospects

The main limitation of this study is the small sample size, which reduces the statistical power and generalizability of the results. However, recruiting patients with FM and IBS is a challenge that has been well documented in the literature (Erdrich et al., 2020; Cardenas-Rojas et al., 2021): Clinical complexity, the need to meet multiple diagnostic criteria, a high burden of symptoms, and frequent reluctance to participate in research studies hinder recruitment in this population. This difficulty is also reflected in our study, in which only 33 of the 78 women who underwent eligibility screening completed the baseline assessments. Logistical obstacles, withdrawal of consent, and, above all, the fear of clinical exacerbations highlight the operational challenges in recruiting and retaining patients with this comorbidity in research programs.

Furthermore, the cross-sectional design precludes causal inference and limits assessment of longitudinal responses to exercise. Despite these limitations, given the current state of knowledge, the present data provide one of the first multidimensional baseline characterizations of women with FM+IBS comorbidities, stratified by disease severity. Future longitudinal studies on larger cohorts, incorporating objective monitoring of physical activity and standardized psychological assessments, are needed to validate and expand upon the clinical patterns identified in the present study.

Several further limitations should be acknowledged. FIQR-based grouping and several of the outcomes tested (pain, physical function) are not fully independent, since FIQR itself incorporates these constructs; this circularity is mitigated, but not eliminated, by our adoption of continuous FIQR as the principal exposure. Categorizing FIQR into three strata necessarily discards information relative to the continuous score, and the three categorical groups are small and unequal in size (n=7, 13, 13); several categorical comparisons, including the Sit-to-Stand test, did not survive Welch’s ANOVA or Kruskal-Wallis testing despite an initially significant standard ANOVA result. Categorical inference is fragile with samples this small. Formal testing was not corrected for multiple comparisons across the roughly 20 outcomes originally examined; all p-values in this manuscript should be interpreted as exploratory. We could not formally adjust for concomitant medication use (analgesics, antidepressants, neuromodulators, hypnotics, gastrointestinal-active agents) given the sample size, despite these classes’ plausible influence on pain, fatigue, sleep, mood, and physical performance. This study did not include FM-only, IBS-only, or healthy comparison groups, and therefore cannot establish FM+IBS as a distinct clinical phenotype relative to either condition in isolation. Floor and ceiling effects are plausible for several instruments (e.g., the 30-second ceiling on the Single Leg Stance Test) and may have attenuated observed associations. This is a single-center, women-only sample from Southern Italy, which limits generalizability to other populations and to men, who also experience FM and IBS. Our dataset retained only the FIQR total score; domain-level scores (physical function, overall impact, symptom intensity) were not available for separate analysis, precluding a more granular examination of which FIQR components drive the associations reported here. Finally, IBS duration was not recorded separately from FM duration, and menopausal status and IBS subtype were not systematically captured; these should be incorporated into future protocols in this population.

## 7 Conclusions

This exploratory study suggests that, in women with FM+IBS comorbidity, higher FM severity is not associated with uniform deterioration across all clinical domains: pain, lower-limb functional capacity, kinesiophobia, and mental health-related quality of life were associated with FIQR scores, whereas gastrointestinal burden, flexibility, and postural balance did not show a clear association in this sample. The observed pattern suggests that FIQR and gastrointestinal symptom burden may not change in parallel, although the estimate was imprecise and requires confirmation in a larger sample. Therefore, future exercise-prescription research should consider the possibility of integrating multiple, only partly correlated domains, while acknowledging that the present cross-sectional, single-arm study cannot itself validate any specific prescriptive approach.

Despite the limitations detailed above, this study offers a descriptive, hypothesis-generating characterization of this clinically complex and understudied population, which may help inform the design of future, adequately powered and appropriately controlled studies of exercise protocols in FM+IBS.

## Data Availability

The datasets presented in this study can be found in online repositories. The names of the repository/repositories and accession number(s) can be found below: 10.6084/m9.figshare.32802620.
