# Supplementary material for: Clinical, functional, and quality-of-life characteristics of women with comorbid fibromyalgia and irritable bowel syndrome and their associations with FIQR scores
Source: Front Physiol. 2026 Oct 1;17:1922718. doi: 10.3389/fphys.2026.1922718 (PMC13668510; doi:10.3389/fphys.2026.1922718)
Supplement: Supplementary file 1 [file Table1.docx]

Supplementary Table S1

**SF-36 eight-domain scores stratified by FM severity group (FIQR), and association with continuous FIQR total score.**

| **SF-36 domain** | **Remission/Mild (n=7)** | **Moderate (n=13)** | **High Severity (n=13)** | **ANOVA p** | **Welch p** | **KW p** | **rho** | **95% CI (bootstrap)** | **p (cont.)** |
| --- | --- | --- | --- | --- | --- | --- | --- | --- | --- |
| Physical Function | 67.1 ± 25.6 | 60.4 ± 13.1 | 38.5 ± 17.6 | **0.002** | **0.007** | **0.004** | -0.64 | [-0.86, -0.31] | **<0.001** |
| Role-Physical | 28.6 ± 36.6 | 9.6 ± 16.3 | 25.0 ± 36.8 | 0.309 | 0.255 | 0.390 | -0.14 | [-0.47, 0.23] | 0.429 |
| Bodily Pain | 44.1 ± 11.2 | 27.6 ± 13.9 | 21.5 ± 9.3 | **0.001** | **0.002** | **0.003** | -0.66 | [-0.82, -0.39] | **<0.001** |
| General Health | 45.9 ± 16.8 | 27.6 ± 14.3 | 23.4 ± 8.9 | **0.003** | **0.020** | **0.012** | -0.45 | [-0.73, -0.07] | **0.010** |
| Vitality | 42.1 ± 19.1 | 25.8 ± 13.4 | 16.5 ± 11.8 | **0.002** | **0.017** | **0.009** | -0.57 | [-0.76, -0.28] | **0.001** |
| Social Functioning | 60.7 ± 19.7 | 34.6 ± 16.3 | 33.7 ± 22.5 | **0.013** | **0.022** | **0.026** | -0.52 | [-0.78, -0.16] | **0.002** |
| Role-Emotional | 38.1 ± 40.5 | 25.6 ± 33.8 | 28.2 ± 32.9 | 0.743 | 0.795 | 0.776 | -0.19 | [-0.51, 0.19] | 0.302 |
| Mental Health | 54.3 ± 12.6 | 43.7 ± 18.7 | 35.7 ± 18.4 | 0.091 | 0.053 | 0.061 | -0.40 | [-0.65, -0.10] | **0.020** |

*Continuous variables are reported as mean ± SD. All eight SF-36 domain scores range from 0–100, with higher scores indicating better health-related quality of life. ANOVA, Welch’s ANOVA, and Kruskal-Wallis (KW) p-values refer to the three-group categorical comparison (Remission/Mild, Moderate, High Severity); rho, 95% CI, and p (cont.) refer to the Spearman correlation between each domain and continuous FIQR total score, with 95% confidence intervals obtained by bootstrap resampling (5,000 resamples, percentile method), consistent with the primary analytical approach used elsewhere in this manuscript (Table 2a). Bold values indicate p<0.05. FM: fibromyalgia; FIQR: Revised Fibromyalgia Impact Questionnaire; SF-36: 36-Item Short-Form Health Survey; KW: Kruskal-Wallis.*
